# The incidence rate over 10 years of naturally occurring, cancer related mutations in the basal core promoter of hepatitis B virus

**DOI:** 10.1016/j.meegid.2015.07.020

**Published:** 2015-08

**Authors:** Xue-Yan Wang, Tim J. Harrison, Qin-Yan Chen, Hai Li, Guo-Jian Li, Mo-Han Liu, Li-Ping Hu, Chao Tan, Qing-Li Yang, Zhong-Liao Fang

**Affiliations:** a Guangxi Zhuang Autonomous Region Center for Disease Prevention and Control, Guangxi Key Laboratory for the Prevention and Control of Viral Hepatitis, Nanning, Guangxi 530028, China; b Division of Medicine, UCL Medical School, London, UK; c Department of Public Health of Guangxi Zhuang Autonomous Region, 35 TaoYuan Road, Nanning, Guangxi 530021, China; d School of Preclinical Medicine, Guangxi Medical University, 22 ShuangYong Road, Nanning, Guangxi 530021, China

**Keywords:** Hepatitis B virus, Basal core promoter, Mutations, Incidence, Genotypes

## Abstract

Cross-sectional analyses showed that the prevalence of basal core promoter (BCP) double mutations (nt 1762T, 1764A) of hepatitis B virus (HBV) gradually increases with age. We aimed to determine the incidence rate of the mutations over 10 years. Study subjects were selected from the Long An cohort established in 2004, including 59 with HBV with single mutations at nt 1762 or 1764 in the BCP and 342 with wild type BCP sequences at baseline. Their serum samples for analysis were obtained at the 3rd and 10th annual visits, respectively. The results showed that the annual incidence rate of BCP double mutations is 3.8% (95% confidence interval [CI]: 1.4–6.2) and tends to decrease with age. The peak incidence is in the 30–34 years age-group. The incidence rate in HBeAg positive individuals (5.5%) is significantly higher than in those without HBeAg (3.4%) (*P* < 0.05). The incidence rate is significantly higher in genotype C (4.8%) than in genotype B (2.8%) or I (3.1%). The incidence rate of the mutations (6.8%) developing from a single mutation at nt 1762 or 1764 is significantly higher than that (3.8%) from the wild type sequence (*P* < 0.005). The difference in incidence of single mutations between nt 1762 (0.7%) and 1764 (0.03%) is significant (*P* < 0.05). In conclusion, the incidence rate of BCP double mutations tends to decrease with age after the age of 35 years. Viruses with a single mutation at nt 1762 or 1764 are more prone to develop double mutations. Nt 1762 is the more common site of the first mutation.

## Highlights

• The annual incidence rate of the basal core promoter (BCP) double mutations is 3.8%.
• The incidence rate tends to decrease with age and the peak appeared early in the life.
• Nucleotide (nt) 1762 is the favoured site of the first mutation.
• Viruses with a single mutation at nt 1762 or 1764 are more prone to develop double mutations.

## 1 Introduction

HBV has a circular, partially double-stranded DNA genome of about 3200 nt with four open reading frames (ORFs), namely the core/precore, polymerase, surface and X ORFs (Tiollais et al., 1985). Transcription of the four ORFs is controlled by the core, large surface, major surface and X promoters, respectively. The core promoter, located between nt 1575 and 1849, consists of the basal core promoter (BCP) (nt 1743–1849) and the upper regulatory region (URR, nt 1613–1742), the latter containing both positive and negative regulatory elements that modulate promoter activity. The core promoter plays a central role in HBV replication and morphogenesis, directing the transcription of both species of 3.5 kb mRNA: pregenomic RNA (pgRNA) and precore mRNA (pre-C mRNA) (Kramvis and Kew, 1999, Quarleri, 2014).

The lack of proof-reading during reverse transcription of the pregenomic RNA favours the development of sequence variants during long-term HBV replication (Harrison, 2006). One of the most critical changes is the appearance of the double mutations at nt 1762 (A → T) and 1764 (G → A) in the BCP. These double mutations, which result in a decrease in the levels of expression of HBeAg and an increase in viral DNA replication (Buckwold et al., 1996, Scaglioni et al., 1997), were first described in the core promoter of HBV from Japanese patients (Sugai et al., 1994, Sato et al., 1995). Subsequently, we and others found that the core promoter double mutations are associated with progressive liver diseases, including hepatocellular carcinoma (HCC) (Nagasaka et al., 1998, Fang et al., 1998, Fang et al., 2002, Kao et al., 2003). The association between the double mutations and HCC has been confirmed by prospective studies (Fang et al., 2008a, Yuen et al., 2009, Chu et al., 2012).

Cross-sectional analyses showed that the prevalence of BCP double mutations gradually increases with age (Nie et al., 2012, Yan et al., 2012). However, data regarding the rate of naturally occurring mutations remain lacking. Although there are a few reports of the development of the double mutations during HBeAg seroconversion, these focused only on the time around the seroconversion of HBeAg (Nie et al., 2012, Chen et al., 2007), which may result in a biased interpretation of the rate of double mutations because BCP double mutations occur more frequency during HBeAg seroconversion (Yuen et al., 2002, Yamaura et al., 2003, Ni et al., 2004).

In this study, based on the Long An cohort (Fang et al., 2008a), we determined the incidence rate of naturally occurring BCP double mutations in HBsAg asymptomatic carriers over 10 years. Meanwhile, we also determined the incidence rate of single mutations (T1762 or A1764) and the incidence rate of double mutations developing from a single mutation (T1762 or A1764).

## 2 Materials and methods

### 2.1 Study subjects and sample design

In order to determine the value of screening carriers of hepatitis B surface antigen (HBsAg) for virus with BCP double mutations as a marker of an extremely high risk of developing HCC, a cohort of 2258 hepatitis B surface antigen positive subjects aged 30–55 was recruited in Guangxi, China early in 2004. This cohort included 1261 subjects with BCP double mutations and 997 with the wild type BCP. We followed the study subjects for three years from 1st July, 2004 (Fang et al., 2008a). Each study subject completed a one-page questionnaire at the first visit and provided a serum sample every six months for the assessment of virological parameters and AFP concentrations, and was monitored for HCC by ultrasonography (US). Then, we followed up for HCC cases but without blood samples drawn every year. We followed up this cohort early in 2014 and collected serum samples again. The study subjects for this study were selected from subjects available for the 10th year visit. The selection criterion is that they were infected with HBV with wild type sequence of BCP at baseline.

In additional, when we established the Long An cohort in 2004, we found some were infected with HBV with a single mutation at nt 1762 or 1764 in BCP. Although they were excluded from the Long An cohort, as controls, we followed up them for three years. Serum samples collected from these subjects during the last visit in the 3rd year were also included in this study.

Informed consent was obtained from each individual. The study protocol conformed to the ethical guidelines of the 1975 declaration of Helsinki and has been approved by the Guangxi Institutional Review Board.

### 2.2 Baseline serological testing and HBV DNA amplification and nucleotide sequencing

The baseline sera testing for HBsAg, HBeAg/anti-HBe, alanine aminotransferase (ALT) levels and HBV core promoter amplification and nucleotide sequencing have been reported previously (Fang et al., 2008a).

### 2.3 Nested PCR for HBV DNA and nucleotide sequencing

DNA was extracted from 85 μl serum by pronase digestion followed by phenol/chloroform extraction. The method to amplify and sequence BCP regions is the same as previously report (Fang et al., 2008a). To amplify the S region, the first round polymerase chain reaction (PCR) was carried out in a 50 μl reaction using primers MD14 (nt 418–433, 5′- GCGCTGCAGCTATGCCTCATCTTC-3′) and HCO2 (nt 761–776, 5′-GCGAAGCTTGCTGTACAGACTTGG-3′) with 5 min hot start followed by 35 cycles of 94 °C for 45 s, 45 °C for 45 s, and 72 °C for 120 s. Second round PCR was carried out on 5 μl of the first round products in a 50 μl reaction using primers ME15 (nt 455–470, 5′-GCGCTGCAGCAAGGTATGTTGCCCG-3′) and HDO3 (nt 734–748, 5′-GCGAAGCTTCATCATCCATATAGC-3′) with 5 min hot start followed by 30 cycles of 94 °C for 45 s, 55 °C for 45 s, and 72 °C for 120 s. Products from the second round were confirmed by agarose gel electrophoresis. HBV DNA positive products were sent to The Sangon Biotech (Shanghai, China) for sequencing using a BigDye Terminator V3.1 Cycle Sequencing kit (Applied Biosystems, Foster City, USA) with sequencing primer ME15.

### 2.4 Measurement of viral loads

Viral load measurements were carried out as described by Garson et al. (Garson et al., 2005). Briefly, HBV DNA was extracted from serum samples using a Qiagen BioRobot 9604 and QIAamp96 Virus Kit reagents (Qiagen, Hilden, Germany). Viral DNA was amplified and quantified in an ABI Prism 7000 sequence detection system (Applied Biosystems, Foster City, CA, USA) using HBV primers and a dual labeled TaqMan probe as described.

### 2.5 HBV genotyping

HBV genotypes were determined using the sequences above and the NCBI Genotyping Tool (http://www.ncbi.nlm.nih.gov/projects/genotyping/formpage.cgi) and the STAR program (http:// www.vgb.ucl.ac.uk/starn.shtml) (Myers et al., 2006).

### 2.6 Statistical analysis

The 95% confidence limits (CI) for the incidence rate of HBV mutations were estimated. Viral loads are presented as median (range). Variables were compared between groups using the chi-square test. Logistic regression analysis was carried out to identify factors that affect the development of BCP double mutations. All *P*-values were two-tailed and *P* < 0.05 was considered to be significant. All statistical analyses were performed using the SPSS software (ver.16.0; Chicago, IL, USA).

## 3 Results

### 3.1 Baseline characteristics

The study subjects consisted of 342 HBsAg positive asymptomatic individuals, including 183 males and 159 females. The youngest and oldest were 40 and 65 years old. The average age (mean ± SD) was 50.1 ± 6.3 years. The average ages of males and females were 48.9 ± 6.0 and 51.5 ± 6.3 years, respectively. One hundred and five (30.7%) study subjects were HBeAg-positive and 237 (69.3%) were HBeAg-negative. Three genotypes were identified, B, C and I, with a prevalence of 41.2%, 47.4% and 11.4% respectively. None of them had received antiviral therapy.

### 3.2 The incidence rate of BCP double mutations

**Fig. 1:**
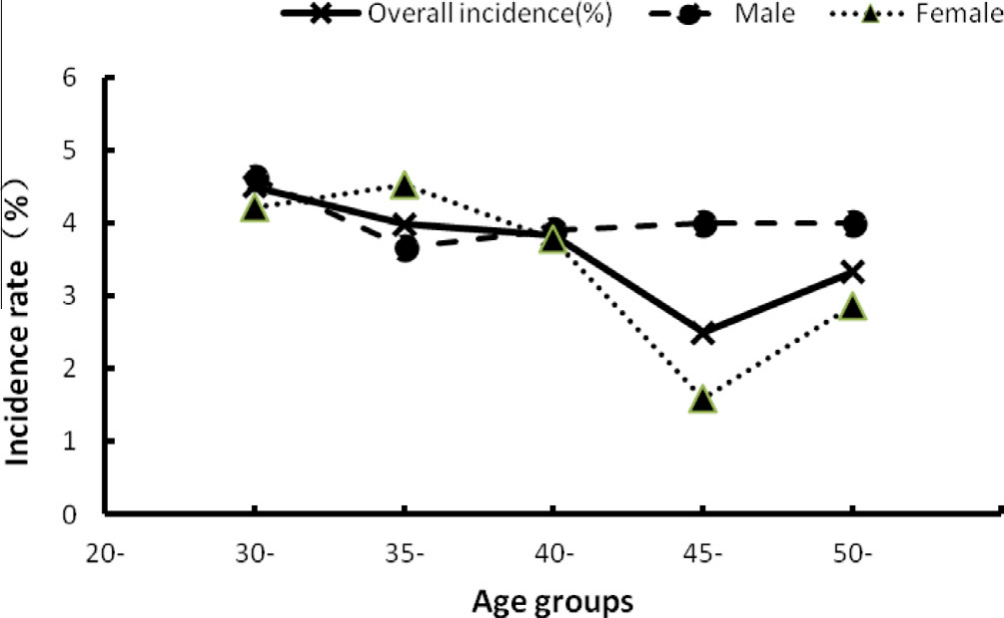
The incidence rate of BCP double mutations, by age.

One hundred and twenty-nine of 342 individuals infected with HBV with wild type BCP sequences at baseline developed double mutations after 10 years, giving the overall annual incidence of 3.8% (95% confidence interval [CI]: 1.4–6.2). The incidence rates of the double mutations in males and females are 3.9% (95% CI: 1.1–6.7) and 3.5% (95% CI: 0.7–6.4), respectively (Table 1); this difference is not significant (*X*^2^ = 0.70, *P* > 0.05). The incidence rate of BCP double mutations trends to decrease with age and the highest was seen in the youngest age group (30–34 years old at baseline) (Fig. 1).

### 3.3 Single mutation at nt 1762 or 1764 are intermediates in the development of the double mutations

At baseline, there were fifty-nine individuals with single BCP mutation, 22 with nt 1762 A → T and 37 with nt 1764 G → A. After three years of follow-up, those with a single mutation at nt 1762 at baseline had a higher incidence of double mutations than those with nt 1764 G → A (9.1% vs. 5.4%), although the difference is not significant (*X*^2^ = 0.4704, *P* > 0.05). The overall annual incidence rate of BCP double mutations of the 59 subjects is 6.8% (95% CI: 0.4–13.2), which is significantly higher than the annual incidence of 3.8% of the double mutations developing from the wild type BCP sequence (*X*^2^ = 21.566, *P* < 0.05), suggesting that a single mutation at 1762 or 1764 is a step to the double mutation (Table 2).

Twenty-four of the 342 individuals developed an A → T point mutation at nt 1762 during the ten years, giving the annual incidence of 0.7% (95% CI: −0.2 to 1.6). However, only one of the 342 developed a G → A point mutation at nt 1764, giving the annual incidence of 0.03% (95% CI: −0.2 to 0.2). The difference in incidence of single mutations between nt 1762 and 1764 is significant (*X*^2^ = 21.96, *P* < 0.05), suggesting that nt 1762 is the more common site of the first mutation (Table 3).

The data above show that single mutations at nt 1762 or 1764 developed from wild type of BCP and BCP double mutations developed later. However, these events occurred in different individuals. In order to determine whether these events may occur in the same individuals, twenty samples from subjects who were found to have developed BCP double mutations by the 10th years were randomly selected from the samples drawn at the 3rd year to determine the sequence of the BCP. The results showed that two of them already had developed BCP double mutations. Five of them had single mutations, including three with 1762T and two with 1764A. The remainder had wild type BCP sequences of at the 3rd year. These data suggested that the single mutation of nt 1762 or 1764 are intermediates in the development of the double mutations (Table 4).

### 3.4 The incidence rate of double mutations and viral loads

The distribution of viral loads was skewed (median 3.09 × 10^4^ IU/mL, range 0–2.64 × 10^9^ IU/mL). The median viral load at baseline is significantly higher in those who developed BCP double mutations after ten years than those who did not develop the mutations (median 189,000 versus 21,234 IU/mL, *P* = 0.02). The incidence rate of BCP double mutations is significantly higher in those with viral loads ⩾10^5^ IU/mL than those with viral loads <10^5^ IU/mL (*X*^2^ = 8.141, *P* < 0.05). These data suggest that the development of BCP double mutations is associated with viral loads at baseline and viral loads ⩾10^5^ IU/mL in serum may be a threshold to predict the likely development of BCP double mutations.

### 3.5 The incidence rate of double mutations and HBeAg

The incidence rate of BCP double mutations in HBeAg positive individuals (55.2%, 95% CI: 45.3–65.1) is significantly higher in those who are HBeAg negative (34.4%, 95% CI: 28.1–40.7) (*X*^2^ = 12.02, *P* < 0.05). The rate of HBeAg seroconversion of fifty-three individuals who developed BCP double mutations after ten years is 75.5% (40/53). Clearly, about one quarter of individuals who developed BCP double mutations remained HBeAg positive, consistent with the fact that the double mutations decrease but not stop expression of HBeAg (Buckwold et al., 1996, Scaglioni et al., 1997).

### 3.6 The incidence rates of double mutations and genotypes

The annual incidence rates of BCP double mutations in genotypes B, C and I are 2.8% (95% CI: 0.1–5.5), 4.8% (95% CI: 1.5–8.1) and 3.1% (95% CI: −2.3 to 8.5), respectively. The differences in the incidence of BCP double mutations between genotypes C and B, and C and I, are *X*^2^ = 13.351 and *X*^2^ = 3.840, respectively, both *P* < 0.05. But the difference in the incidence of BCP double mutations between genotypes B and I are not significant (*X*^2^ = 0.145, *P* > 0.05). These data suggest that genotype C is associated with the development of BCP double mutations (Table 5).

### 3.7 Analysis for factors associated with the development of BCP double mutations

Multivariable logistic regression analysis was carried out to identify factors that affect the development of BCP double mutations. The independent variables included sex, age, viral loads, HBeAg, genotypes and ALT. On univariate analysis, viral loads, genotypes and HBeAg status were independently associated with the development of BCP double mutations but sex, age and ALT were not. On multivariate analysis, genotype C was associated with the development of BCP double mutations (OR = 2.253, *P* = 0.001) (Table 6).

## 4 Discussion

To our knowledge, this is the first study to report the annual incidence rate of naturally occurring, cancer related mutations in the basal core promoter of hepatitis B virus. The major findings of the study are that the annual incidence rate of BCP double mutations is 3.8%. The incidence rate of BCP double mutations trends to decrease with age after the age of 35 years and the highest was seen in the youngest age group (30–34 years group) in the cohort. The single mutations nt 1762 and 1764 are intermediates in the development of the double mutations and nt 1762 is the more common site of the first mutation. Genotype C and HBeAg status are associated with the development of BCP double mutations. The strength of this study is that the data are derived from a long-term prospective study, which makes them very relevant. A weakness of this study is that the study subjects did not include individuals younger than 30 years old, so we could not obtain the incidence rate of BCP double mutations for these age groups. Another weakness is that we did not sequence the PreS1/S2 region that has been reported to be associated with the development of HCC (Fang et al., 2008b), so we could not obtain the incidence rate of this region.

It has been reported from cross-sectional studies that the prevalence of BCP double mutations increased with age (Fang et al., 2008a, Nie et al., 2012, Yin et al., 2010). The prevalence is about 6–8% in those aged below 15 years old (Yin et al., 2010, Kang et al., 2011) and about 25–69% in adults (Yuen et al., 2002, Yin et al., 2010). However, the annual incidence rate of BCP double mutations remains unclear. There are a few studies regarding the development of BCP double mutations (Chen et al., 2007, Nie et al., 2012), unfortunately, most studies have focused on the time around seroconversion of HBeAg (Chen et al., 2007, Nie et al., 2012). BCP double mutations occur more frequently during HBeAg seroconversion (Yuen et al., 2002, Yamaura et al., 2003, Ni et al., 2004). Therefore, these studies may result in a biased mutation rate of the double mutations. In this study, we included both HBeAg-positive and -negative study subjects, with a long-term follow up. We found that BCP double mutations occurred in both HBeAg-positive and -negative study subjects, although the rate in individuals with HBeAg is significantly higher than in those who are HBeAg negative. Furthermore, our study subjects are all HBsAg asymptomatic carriers and have not received antiviral therapy. Therefore, our results more likely reflect the natural rate of development of BCP double mutations.

The causal relationship of HCC and the double mutations has been confirmed by prospective studies (Fang et al., 2008a, Yuen et al., 2009, Chu et al., 2012). The incidence of HCC increases with age (Yang and Roberts, 2010, Nordenstedt et al., 2010). In Guangxi, China, the peak age of diagnosis with HCC is 45–49 years (Zhang et al., 1997). We found that the incidence rate of BCP double mutations is highest in age group of 30–34, which confirmed our previous finding from a cross-sectional study (Fang et al., 2008a) that the prevalence of BCP double mutations is highest in the group aged 40–44. It is reasonable to speculate that BCP double mutations developed about 10 years before the development of HCC. Therefore, our findings are important to understand the mechanisms of oncogenesis of BCP double mutations.

BCP double mutations constitute a typical pattern of mutations in the core promoter and are seen most frequently, while single mutations at nt 1762 and 1764 are rarely detected in HBV patients (Sugai et al., 1994, Buckwold et al., 1997). It is unclear whether the development of BCP double mutations is via a single mutation. In this study, we found that single mutations at nt 1762 or 1764 developed from the wild type BCP and then progressed to BCP double mutations, suggesting that the single mutations at nt 1762 or 1764 are intermediates in the development of the double mutations. Furthermore, we found that nt 1762 is the favoured site for the initial mutation. HBeAg is not required for viral replication (Tong et al., 1991). However, the loss of HBeAg may be a sign of immune escape (Carman et al., 1989). The nt 1762T mutation can suppress precore RNA transcription, which decreases the production of HBeAg and increases the efficiency of progeny virus synthesis. In contrast, the nt 1764A mutant does not suppress precore RNA transcription but reduces slightly the efficiency of virus progeny synthesis (Buckwold et al., 1997). Therefore, it is possible that nt 1762 develops mutation first under immune pressure.

Data from cross-sectional studies showed that BCP double mutations are associated with genotype. Genotypes C and D have a higher frequency of BCP double mutations than genotypes A and B (Kramvis and Kew, 1999, Quarleri, 2014). These finding were confirmed by our prospective cohort study. We found that the annual incidence rate of BCP double mutations in genotype C is significantly higher than that of genotype C or I.

It has been reported that the double mutations decrease but do not stop expression of HBeAg in cell culture (Buckwold et al., 1996, Scaglioni et al., 1997). Cross-sectional studies also showed that not all of individuals with BCP double mutations were negative for HBeAg but the titer of HBeAg is lower (Qin et al., 2009, Yim et al., 2015). We found that about one quarter of individuals remain positive for HBeAg despite having double mutations in the BCP.

In conclusion, the incidence rate of BCP double mutations tends to decrease with age after the age of 35 years. Viruses with a single mutation at nt 1762 or 1764 are more prone to develop double mutations. Nt 1762 is the more common site of the first mutation.

## Figures and Tables

**Table 1:** The incidence rate of BCP double mutations, by gender.

Gender	Number	Number of double mutations	Incidence rate (%)	Annual incidence rate (%) (95% CI)
Male	183	73	38.9	3.9 (1.1–6.7)
Female	159	56	35.2	3.5 (2.9–6.4)
Total	342	129	35.7	3.6 (1.6–5.6)

The differences in the incidence rate of BCP double mutations between males and females is *X*^2^ = 0.790, *P* > 0.05.

**Table 2:** The incidence rate of BCP double mutations developing from single mutations over three years.

Type of single mutation at baseline	Number	Progressed to double mutations (nt 1762T, nt 1764A) at third year	Annual incidence rate (%)
nt 1762T	22	6	9.1 (−2.9 to 21.1)
nt 1764A	37	6	5.4 (−1.9 to 12.7)
Total	59	12	6.8 (0.4 to 13.2)

The differences in the incidence rate of BCP double mutations between nt 1762T and nt 1764A is *X*^2^ = 0.4704, *P* > 0.05.

**Table 3:** The incidence rate of single mutations developing from wild type BCP sequences over ten years.

Gender	No.	nt 1762A → T mutation	Annual incidence rate (%) (95% CI)	nt 1764G → A mutation	Annual incidence rate (%) (95% CI)	nt 1762A → T or1764G → A mutation	Annual incidence rate (%) (95% CI)
Male	183	14	0.8 (−0.5 to 2.1)	0	0	14	0.8 (−0.5 to 2.1)
Female	159	10	0.6 (−0.6 to 1.8)	1	0.06 (−0.3 to 0.4)	11	0.7 (0.6–2.0)
Total	342	24	0.7 (−0.2 to 1.6)	1	0.03 (-0.2 to 0.2)	25	0.7 (−0.2 to 1.6)

**Table 4:** Characteristics of individuals developing BCP double mutations from the wild type sequence via a single mutation.

Samples	Gender^▾^	Ages	Genotypes	Viral loads (IU/ml)	HBeAg	ALT^∗^ IU/ml)	BCP sequence at		
Baseline^#^	3rd year^#^	10th year^#^
CZ238	F	40	C	2.51E + 08	+	−	W	1762T	M
DB253	M	44	B	5.17E + 07	+	285	W	1762T	M
DJ017	F	40	C	2.80E + 06	+	−	W	1762T	M
DJ053	F	37	B	78.2	−	−	W	W	M
DW454	F	36	C	1.16E + 08	+	−	W	W	M
DW456	M	34	B	22,698	−	−	W	W	M
GA024	M	31	I	3.04E+08	+	−	W	W	M
GM174	M	42	B	8.32E+07	+	−	W	W	M
GY43	F	52	B	8.80E+07	+	−	W	W	M
JD171	M	35	C	1.10E+07	+	59	W	W	M
ND128	F	35	C	8.16E+08	+	−	W	W	M
NX109	M	35	I	6.20E+08	+	−	W	M	M
TJ163	M	36	C	3.66E+08	+	−	W	M	M
TM083	M	35	C	5.90E+08	+	−	W	W	M
TS092	F	40	B	1.92E+08	+	−	W	1764A	M
TX72	F	35	C	9.40E+07	−	66	W	W	M
WX288	M	42	C	5.00E+08	+	−	W	W	M
XW64	F	35	C	6.31E+08	+	−	W	W	M
YL340	F	35	B	3.51E+08	+	−	W	1764A	M
YY416	F	42	C	3.72E+06	+	−	W	W	M

▾: M, Male; F, Female. ∗: ALT: alanine aminotransferase, “−”, normal ALT, the cut-off is 40 IU/ml. #: W, wild type, M: BCP double mutations.

**Table 5:** The incidence rate of BCP double mutations by genotype.

Gender	Number	Number of double mutations	Incidence rate (%)	Annual incidence rate (%) (95% CI)
Genotype B	141	39(9)	27.7	2.8 (0.1–5.5)
Genotype C	162	78(12)	48.2	4.8 (1.5–8.1)
Genotype I	39	12(4)	30.8	3.1 (−2.3 to 8.5)
Total	342	129(25)	37.7	3.8 (1.8–5.8)

The differences in the incidence of BCP double mutations between genotypes C and B, and C and I, are *X*^2^ = 13.351 and *X*^2^ = 3.840, respectively, both *P* < 0.05. The difference in the incidence of BCP double mutations between genotypes B and I is not significant (*X*^2^ = 0.145, *P* > 0.05).

**Table 6:** Logistic regression analysis for factors associated with the development of BCP double mutations.

Analysis models	Variables	*p* Value	Hazard ratio	95% CI for hazard ratio	
Lower	Upper
Univariate analysis	Sex				
Female^∗^				
Male	0.374	1.221	0.786	1.895
Ages				
30–34^∗^				
35–40	0.511	0.809	0.430	1.523
40–45	0.423	0.758	0.385	1.492
45–50	0.024	0.355	0.144	0.872
50-	0.315	0.670	0.307	1.462
Genotypes				
Genotype B^∗^				
Genotype C	0.00	2.429	1.502	3.928
Genotype I	0.703	1.162	0.536	2.520
Viral loads				
No^∗^				
⩾10^5^ IU/ml	0.006	1.870	1.199	2.917
HBeAg				
Negative^∗^				
Positive	0.001	2.159	1.350	3.454
ALT▴				
<40 IU/ml				
⩾40	0.773	1.087	0.616	1.918
Multivariate analysis	Genotypes				
Genotype B^∗^				
Genotype C	0.001	2.253	1.381	3.676
Genotype I	0.848	1.080	0.492	2.368
HBeAg (+)	0.003	2.070	1.277	3.354

^*^The variable used for comparison; ALT, ▴Alanine aminotransferase.
